# Copan eNAT Transport System To Address Challenges in COVID-19 Diagnostics in Regions with Limited Testing Access

**DOI:** 10.1128/JCM.00110-21

**Published:** 2021-04-20

**Authors:** Melissa Richard-Greenblatt, Courtney E. Comar, Laurence Flevaud, Marina Berti, Rebecca M. Harris, Susan R. Weiss, Laurel Glaser

**Affiliations:** a Department of Pathology and Laboratory Medicine, Perelman School of Medicine, University of Pennsylvania, Philadelphia, Pennsylvania, USA; b Clinical Microbiology Laboratory, Hospital of the University of Pennsylvania, Philadelphia, Pennsylvania, USA; c Infectious Disease Diagnostics Laboratory, Children’s Hospital of Philadelphia, Philadelphia, Pennsylvania, USA; d Department of Microbiology, Perelman School of Medicine, University of Pennsylvania, Philadelphia, Pennsylvania, USA; e Médicins Sans Frontières (MSF), Barcelona, Spain; UNC School of Medicine

**Keywords:** COVID-19, Cepheid Xpert Xpress SARS-CoV-2, SARS-CoV-2, SARS-CoV-2 inactivation, SARS-CoV-2 specimen stability, eNAT, limited testing access, low-resource settings, point of care, rural healthcare

## Abstract

Community-based health care clinics and hospital outreach services have the potential to expand coronavirus disease 2019 (COVID-19) diagnostics to rural areas. However, reduced specimen stability during extended transport, the absence of a cold chain to centralized laboratories, and biosafety concerns surrounding specimen handling have limited this expansion. In the following study, we evaluated eNAT (Copan Italia, Brescia, Italy) as an alternative transport system to address the biosafety and stability challenges associated with expanding COVID-19 diagnostics to rural and remote regions. In this study, we demonstrated that high-titer severe acute respiratory virus syndrome coronavirus 2 (SARS-CoV-2) lysate placed into eNAT medium cannot be propagated in cell culture, supporting viral inactivation. To account for off-site testing in these settings, we assessed the stability of contrived nasopharyngeal (NP) specimens stored for up to 14 days in various transport media (eNAT, eSwab, viral transport medium [VTM], saline, and phosphate-buffered saline [PBS]) at 4°C, 22 to 25°C, and 35°C. The molecular detection of SARS-CoV-2 was unaffected by sample storage temperature over the 2 weeks when stored in eNAT or PBS (change in cycle threshold, ≤1). In contrast, variable stability was observed across test conditions for other transport media. As eNAT can inactivate SARS-CoV-2, it may support COVID-19 diagnostics at the point of care. Evaluation of compatibility of eNAT with Cepheid Xpert Xpress SARS-CoV-2 assay demonstrated diagnostic accuracy and sensitivity equivalent to those of VTM. Taken together, these findings suggest that the implementation of eNAT as a collection device can expand COVID-19 testing to areas with limited health care access.

## Abstract (precis)

Community-based health care clinics and hospital outreach services have the potential to expand coronavirus disease 2019 (COVID-19) diagnostics to rural areas. However, reduced specimen stability during extended transport, the absence of a cold chain to centralized laboratories, and biosafety concerns surrounding specimen handling have limited this expansion.

## INTRODUCTION

Infrastructure needed to comply with the World Health Organization (WHO) guidelines for diagnostic laboratory workup for severe acute respiratory virus syndrome coronavirus 2 (SARS-CoV-2) infection is limited in many parts of the world. Consequently, testing is often centralized to laboratories based in cities with established biosafety level 2 (BSL-2) facilities. The limited availability of testing facilities in rural and remote regions has contributed significantly to disparities in SARS-CoV-2 testing (1–4). Community-based health care clinics and hospital outreach services have the potential to expand coronavirus disease 2019 (COVID-19) testing in rural areas. However, reduced specimen stability during extended transport, the absence of a cold chain to centralized laboratories, and biosafety concerns during sample collection and transport have limited this expansion (3, 4). Identifying strategies to expand testing to areas with limited health care access is necessary to improve health outcomes and reduce transmission in these communities.

Using an alternative transport system for COVID-19 diagnostics may improve access to laboratory services by enhancing specimen stability and improving biosafety. The preferred specimen for respiratory viruses has been a nasopharyngeal (NP) swab placed in universal viral transport medium (VTM), as the system can preserve virus viability as well as support molecular diagnostics. In recent years, respiratory virus detection has shifted almost entirely from viral culture to nucleic acid testing. The transition in viral diagnostics has provided the opportunity to explore alternative transport medium types for COVID-19 diagnostics. Current Food and Drug Administration (FDA) guidance recommends liquid Amies (eSwab), normal saline, and phosphate-buffered saline (PBS) as alternative transport media for COVID-19 diagnostic testing. However, manufacturers do not report the stability of viral nucleic acid stored in these medium types, and, based on the limited data available, the FDA recommends the storage of specimens for SARS-CoV-2 detection for up to 72 h at 4°C. In addition, limited studies exist that assess alternative transport systems capable of inactivating the SARS-CoV-2 virus, enabling safe transport from rural and remote settings (5, 6). Immediate viral inactivation upon collection may also permit the decentralization of COVID-19 testing from BSL-2-certified laboratories and promote the use of platforms that can be deployed at the point of care (POC) (7).

Inactivation methods involving the immersion of clinical samples in solution containing the denaturant agent, guanidine thiocyanate, were implemented during past Ebola outbreaks to increase testing capacity and reduce exposure risk in the analysis chain (8). In addition to Ebola, other viruses can be inactivated by the agent (9, 10). Copan eNAT (Copan Italia, Brescia, Italy) is an FDA-cleared, commercially available transport system that combines a flocked swab with a guanidine thiocyanate-based medium. The product is claimed to inactivate microorganisms (Gram-positive and Gram-negative bacteria, yeasts, and molds) as well as preserve nucleic acid for molecular testing. However, limited data exist related to the inactivation and nucleic acid stability of viruses collected and stored in eNAT. Therefore, as a potential mechanism to reduce risk associated with specimen handling and increase access to COVID-19 testing, the following study evaluated eNAT as an alternative transport system for SARS-CoV-2 molecular testing. The ability of eNAT to inactivate SARS-CoV-2, maintain viral RNA stability over time at various temperatures (4 to 35°C), and demonstrate compatibility with the Xpert Xpress SARS-CoV-2 assay (Cepheid, CA, USA) were assessed.

## MATERIALS AND METHODS

### Preparation of SARS-CoV-2 stock.

SARS-CoV-2 (USA-WA1/2020 strain) was obtained from BEI (NR-52281) and propagated in African green monkey kidney Vero-E6 cells (ATCC CRL-1586). The Vero-E6 cells were cultured in Dulbecco’s modified Eagle’s medium (DMEM; Gibco catalog no. 11965), supplemented with 10% fetal bovine serum (FBS), 100 U/ml of penicillin, 100 μg/ml streptomycin, 50 μg/ml gentamicin, 1 mM sodium pyruvate, and 10 mM HEPES. SARS-CoV-2 stock (2.3 × 10^7^ PFU/ml; corresponding to a *C_T_* value of 11.5 on a laboratory-developed SARS-CoV-2 assay on the BD MAX system) was made by infecting Vero-E6 cells at a multiplicity of infection of 0.01 in serum-free DMEM for 1 h at 37°C. After 1 h, the inoculum was removed and replaced with 2% FBS DMEM. Cells were incubated for 3 to 4 days at 37°C, and once significant cytopathic effect was observed, the virus stock was harvested. Stock was frozen/thawed one to two times, and then cellular debris was removed by centrifugation. Infectious virus concentration was determined by viral plaque assay as previously described (11). The genomic RNA was sequenced and was determined to have 100% identity with the expected strain (GenBank accession no. MN985325.1).

### Inactivation of SARS-CoV-2 by eNAT.

Evaluation of viral inactivation by Copan eNAT transport medium was performed by preparing mock upper respiratory tract specimens from SARS-CoV-2 stock. Regular-sized flocked swabs were dipped into 100 μl of SARS-CoV-2 stock solution before being placed into a transport tube containing 1 ml of eNAT (eNAT 6C057N.RUO; Copan Italia) or 1 ml of DMEM (positive control). In parallel, a negative control was prepared by dipping a regular-sized flocked swab in 100 μl of DMEM and placing it into a transport tube containing 1 ml eNAT. All three mock specimen types were vortexed and then incubated at room temperature (22 to 26°C) for 10 min. Data were gathered from three independent experiments.

The presence of infectious particles was determined by viral plaque assay (11). Each specimen type was 10-fold serially diluted to 10^−6^, starting with 50 μl of the original sample. The dilutions were plated to Vero E6 cells and incubated for 1 h at 37°C. The inoculum was overlaid with DMEM plus agarose (0.1%) and reincubated for 72 h at 37°C. Cells were fixed with 4% paraformaldehyde and stained with 1% crystal violet. The viral titer of the mock-infected samples was determined by calculating plaque-forming units per milliliter-based plaque counts. All virus manipulations were conducted in a biosafety level 3 laboratory using approved personal protective equipment and protocols.

### Comparison of molecular detection stability of SARS-CoV-2 in alternative transport media stored at various temperatures over 14 days.

Experiments were performed using residual NP swab specimens collected in 3 ml of 0.9% saline previously characterized as SARS-CoV-2 positive in the Hospital of the University of Pennsylvania Clinical Microbiology Laboratory (Xpert Xpress SARS-CoV-2; Cepheid). Five specimens were pooled (1 ml of each specimen) to obtain sufficient volume for the stability studies. Specimens were selected if they were collected <48 h prior to pooling and stored at 2 to 8°C and had a cycle threshold (*C_T_*) value of <30 (*C_T_* range, 18 to 27) to avoid any loss in assay reproducibility when a signal occurred near the limit of detection.

Samples were prepared in triplicate for each condition (4°C, 22 to 25°C, and 35°C) and each transport medium tested. Therefore, a total of 9 samples were contrived for each type of transport media. Transport media evaluated in these experiments included universal VTM (BD Diagnostics, MD, USA), eSwab (Copan Italia, Brescia, Italy), 0.9% saline (prepared saline solution; BD BBL, MD, USA), phosphate-buffered saline (0.067 M, pH 6.8; Hardy Diagnostics, CA, USA), and eNAT (Copan Italia, Brescia, Italy). Each contrived sample was prepared by dipping a regular-sized flocked swab into the freshly prepared pooled saline and placing it into a 15-ml polypropylene conical tube (Corning, AZ, USA) containing 3 ml of transport medium. Due to limited reagent availability and allocations of Cepheid Xpert Xpress SARS-CoV-2 cartridges for our institutions, the stability of specimens for molecular detection of SARS-CoV-2 was measured using a laboratory-developed Emergency Use Authorized reverse transcription-PCR (RT-PCR) assay on the BD Max system (BD Diagnostics, MD, USA). *C_T_* values for SARS-CoV-2 were determined at baseline and compared to values at 1, 3, 7, and 14 day(s) of storage at 4°C, 22 to 25°C, and 35°C. Based on the BD MAX interassay precision (SARS-CoV-2 target *C_T_* range, ±0.7), we considered a change in *C_T_* (Δ*C_T_*) score from baseline (day 0) to be equivalent if ≤1.0 (12, 13) and a loss in stability/sensitivity if an increase in *C_T_* of ≥1.1 was observed. As specimen stability is independent of the molecular platform used, the findings from these studies can be extended to the POC.

### Compatibility of eNAT with Xpert Xpress SARS-CoV-2 assay.

To determine the compatibility of eNAT with the Xpert SARS-CoV-2 assay, we compared the performance of matched eNAT-VTM paired samples for the detection of SARS-CoV-2. Matched specimens were contrived using previously characterized NP swab specimens in 0.9% saline (*n* = 20) collected within 48 h and stored immediately at 4°C following clinical testing. All samples included were from the adult population at the Hospital of the University of Pennsylvania. To ensure the accuracy studies encompassed the Xpert Xpress SARS-CoV-2 assay detection range, all previously characterized positive samples were screened and selected for based on *C_T_* value. Five samples were collected for each of the following *C_T_* ranges: (i) ≤25, (ii) 26 to 29, and (iii) ≥30. Each specimen was prepared by dipping a flocked swab into the clinical specimen and then placing it into 1 ml of the respective medium. Specimens were vortexed and immediately run using the Cepheid Xpert Xpress SARS-CoV-2 assay according to the manufacturer’s guidelines. Since data are not publicly available surrounding interassay variability for the SARS-CoV-2 express assay, we used precision data from the Children’s Hospital of Philadelphia Infectious Diseases Diagnostic Laboratory (*n* = 58). The interassay coefficient of variation was determined to be 1.3% for both targets. *C_T_* ranges of 1.7 and 1.9 were observed for the E gene and N2 gene, respectively. Therefore, we considered any difference between paired specimen *C_T_* values of ≥2.0 to be significant.

Lastly, we investigated the impact of eNAT on the analytical sensitivity of the Xpert Xpress SARS-CoV-2 assay. The assay has a claimed LoD of 250 copies/ml for NP swabs collected in VTM. Based on the reported LoD, a dilution series (25, 125, 250, and 500 copies/ml) was performed in triplicate to determine if eNAT impacts analytical sensitivity. Contrived specimens were prepared for each transport medium. Pooled saline from negative NP swab collections (*n* = 5) was added to eNAT or VTM at a dilution of 1:10. The spiked transport medium was used to serially dilute SARS-CoV-2-positive material (SeraCare Life Sciences Inc., MA, USA). Each sample was run immediately using the Xpert Xpress SARS-CoV-2 assay.

### Statistical analysis.

Analysis for this study was performed using GraphPad Prism version 7.04 (San Diego, CA, USA). A comparison in the sensitivity of collection methods (eNAT and VTM) for detecting SARS-CoV-2 was performed using a paired *t* test. Percent agreement of the collection method was determined based on previous SARS-CoV-2 RT-PCR characterization by the clinical laboratory. Kappa was calculated to quantify the degree of overall agreement between the two transport media for the detection of SARS-CoV-2 using the Xpert Xpress assay.

## RESULTS

### Inactivation of infectious SARS-CoV-2 by eNAT.

Before evaluating the effectiveness of eNAT inactivation of SARS-CoV-2, we investigated the cytotoxic effect of the transport medium on Vero E6 cells. Cell lysis was only observed in the first (10^−1^) of the dilution series. Based on these findings, the limit of detectable virus was 500 PFU/ml.

**FIG 1:**
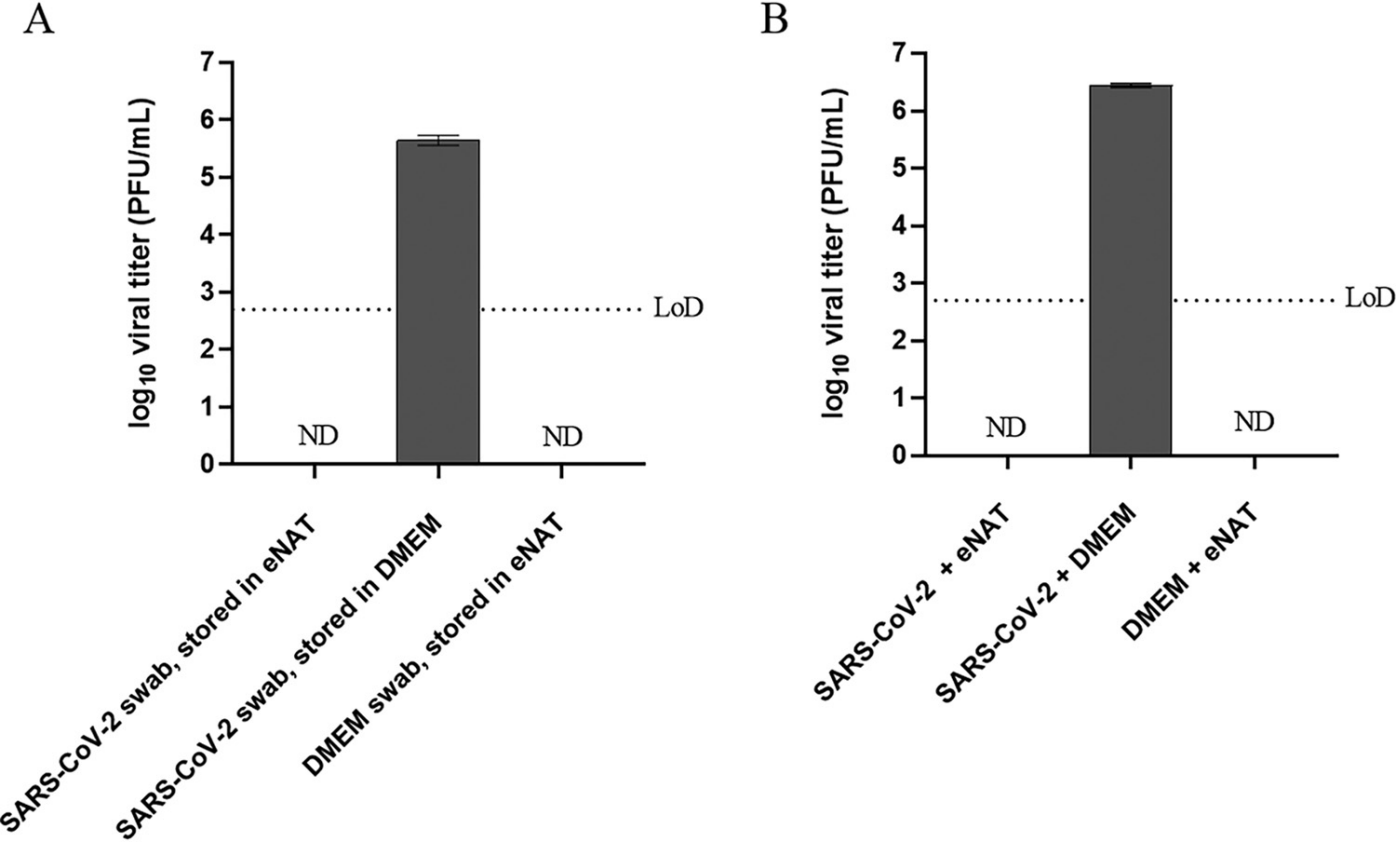
eNAT inactivation of SARS-CoV-2. To determine the efficacy of eNAT for inactivating SARS-CoV-2, infectious SARS-CoV-2 was quantified by viral plaque assay on Vero E6 cells following incubation with eNAT or DMEM for 10 min at room temperature. Analysis of eNAT inactivation was performed using two sample types. (A) Swabs were inoculated with infectious SARS-CoV-2 stock (∼100 μl; stock, 2.3 × 10^7^ PFU/ml) or DMEM and were placed into transport tubes containing 1 ml of eNAT or DMEM. (B) Equal volumes of SARS-CoV-2 stock (2.3 × 10^7^ PFU/ml) or DMEM were combined with eNAT or DMEM (final volume of 200 μl) in the absence of a swab. Bars are representative of experimental triplicates (means ± standard deviations). Data displayed are from a single representative experiment of three independent experiments. The dotted line indicates the limit of detection of 500 PFU/ml due to eNAT lysis of the Vero E6 cells at the lowest dilution (10^−1^). Abbreviations: ND, not detected; LoD, limit of detection; DMEM, Dulbecco’s modified Eagle medium.

The inactivation of infectious SARS-CoV-2 by eNAT was evaluated using contrived specimens to mimic those obtained in the clinical laboratory. Following 10 min of incubation at room temperature, no SARS-CoV-2 could be detected by standard viral plaque assay (Fig. 1A). In contrast, virus-soaked swabs placed in serum-free DMEM had detectable amounts (4.4 × 10^5^ PFU/ml) of infectious SARS-CoV-2 when quantified by plaque assay.

We wanted to further increase the infectious virus concentration and dilution of eNAT. Therefore, an equal volume of SARS-CoV-2 stock was combined with eNAT or DMEM and placed at room temperature for 10 min before quantifying infectious virus particles. Increasing concentrations of infectious SARS-CoV-2, from 2.1 × 10^6^ PFU/ml to 1.2 × 10^7^ PFU/ml, and diluting eNAT 1:1 did not impact its inactivation efficacy (Fig. 1B). Therefore, these findings suggest that specimens collected in Copan eNAT can inactivate infectious SARS-CoV-2 at clinically relevant concentrations.

### Molecular detection stability of SARS-CoV-2 from swabs stored in different transport media at different temperatures.

**FIG 2:**
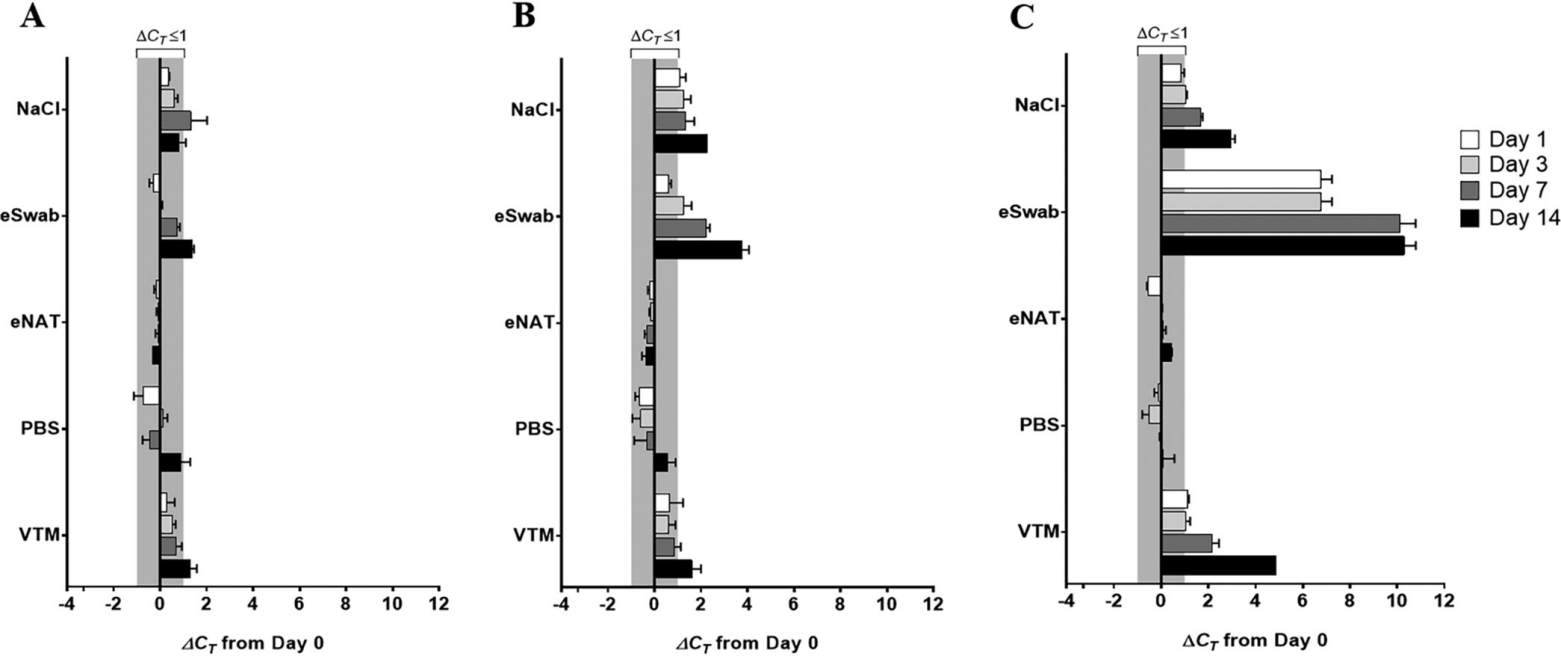
Effect of alternative transport medium storage time and temperature on the molecular detection of SARS-CoV-2 nucleic acid in contrived nasopharyngeal swab specimens. Transport medium was stored at 4°C (A), 22 to 25°C (B), or 35°C (C) for 14 days. SARS-CoV-2 *C_T_* values for each sample were determined at baseline and days 1, 3, 7, and 14. The change in *C_T_* value from baseline (Δ*C_T_*) was calculated and plotted. Bars are representative of experimental triplicates and are presented as mean Δ*C_T_* values ± standard deviations from the baseline. Loss in sample sensitivity from the baseline is plotted as a positive value. Δ*C_T_* values of >1 are considered a significant change from baseline. Abbreviations: NaCl, 0.9% saline; PBS, phosphate-buffered saline; VTM, viral transport medium.

In low- and middle-income countries (LMICs), clinical sample transportation can take >7 days until receipt by the processing laboratories due to a lack of well-established transportation networks (14, 15). These specimens are often transported by motorbike, resulting in challenges in maintaining a cold chain throughout the delivery process. Similar challenges exist in developed countries, where certain delivery methods result in breaks in the cold chain (e.g., mail-in samples), and specimens collected in rural areas may experience delays in transport to centralized laboratories. To address specimen stability challenges related to extended transport times and breaks in the cold chain, we evaluated the molecular detection stability of SARS-CoV-2 stored in eNAT to other transport media currently recommended for collection of upper respiratory tract specimens (Fig. 2). SARS-CoV-2 molecular detection remained stable (Δ*C_T_* < 1) for all transport media when stored at 4°C for the duration of the study period, except for eSwab and VTM. Both transport media demonstrated a decreasing signal over time, which reflected a small (Δ*C_T_* < 2) but significant Δ*C_T_* from baseline at day 14.

At higher storage temperatures (room temperature and 35°C), reduced molecular detection stability was observed for saline, eSwab, and VTM within the 14-day time period. Temperature and length of storage demonstrated the greatest impact on SARS-CoV-2 RNA recovery from the eSwab. A significant loss in detection was observed as early as day 3 and day 1 of storage at 22°C and 35°C, respectively. Storage at 35°C showed the greatest loss in sensitivity, with a *C_T_* change of ∼10 by day 7.

Compared to the eSwab, the effect of higher storage temperatures on SARS-CoV-2 detection from saline and VTM was not as extreme. At both 22°C and 35°C, *C_T_* values for SARS-CoV-2 stored in saline gradually rose above the Δ*C_T_* significance threshold to reach a maximum Δ*C_T_* from baseline of 2.3 ± 0.06 and 3.0 ± 0.2, respectively. A significant loss in detection when stored in VTM at 22°C was observed only on day 14 (Δ*C_T_*, 1.6 ± 0.7); however, at 35°C, a significant loss in detection was observed on both days 7 (Δ*C_T_*, 2.2 ± 0.5) and 14 (Δ*C_T_*, 4.9 ± 0.2).

Storage temperature and time did not appear to have any impact on SARS-CoV-2 detection for NP specimens stored in eNAT or PBS. Therefore, these findings suggest that eNAT and PBS have utility for extended transport and that the reliability of a cold chain is inconsequential when using these two types of transport media.

### Compatibility of eNAT with Cepheid Xpert Xpress SARS-CoV-2.

As we observed eNAT to both inactivate SARS-CoV-2 and maintain viral RNA stability over time at various temperatures (4 to 35°C), it was of further interest to evaluate the compatibility of the transport medium with POC SARS-CoV-2 molecular diagnostics. Due to the wide distribution of the Cepheid GeneXpert System in rural and remote regions globally, we assessed the impact of eNAT on the analytical sensitivity and clinical accuracy of SARS-CoV-2 detection using the Xpert Xpress SARS-CoV-2 assay. Twenty paired eNAT and VTM specimens (positive, *n* = 15; negative, *n* = 5) contrived from previously characterized NP specimens demonstrated 100% overall agreement (20/20; κ = 1.0) using the Xpert Xpress SARS-CoV-2 assay (Table 1). Of the 15 positive specimen pairs tested, none were considered significantly different based on the SARS-CoV-2 cartridge interassay variability, suggesting that eNAT does not impact SARS-CoV-2 detection when using the Xpert Xpress SARS-CoV-2 assay (Table 2).

**TABLE 1:** Accuracy of SARS-CoV-2 detection in 20 contrived eNAT and VTM NP swab sample pairs using the Cepheid Xpert Xpress SARS-CoV-2 assay^*a*^

Result (no.) for indicated transport medium				% agreement (95% CI)	
Total tested	eNAT positive	VTM positive	Total negative	PPA	NPA
20	15	15	5	100 (78.2–100)	100 (47.8–100)

a Abbreviations: VTM, viral transport medium; CI, confidence interval; PPA, positive percent agreement; NPA, negative percent agreement.

**TABLE 2:** Individual *C_T_* values for matched SARS-CoV-2-positive NP swab sample pairs and interassay *C_T_* value differences determined by the Cepheid Xpert Xpress SARS-CoV-2 assay

Sample no.	*C_T_* value				Difference in *C_T_* values	
	E gene		N2 gene			
	eNAT	VTM	eNAT	VTM	E gene	N2 gene
1	20.6	21.2	23.2	24.0	−0.6	−0.8
2	33.2	34.7	36.5	37.0	−1.5	−0.5
3	21.1	21.2	23.6	23.7	−0.1	−0.1
4	26.0	26.3	28.5	28.8	−0.3	−0.3
5	29.8	30.1	31.8	32.8	−0.3	−1.0
6	25.3	23.7	27.4	25.8	1.6	1.6
7	29.0	29.7	30.7	31.8	−0.7	−1.1
8	30.4	30.3	33.2	33.1	−0.1	−0.1
9	30.3	31.1	32.8	33.4	−0.8	−0.6
10	35.0	33.6	36.9	36.3	1.4	0.6
11	18.4	19.2	20.7	21.7	−0.8	−1.0
12	35.1	35.2	38.0	39.4	−0.1	−1.4
13	31.9	32.3	35.2	35.5	−0.4	−0.3
14	31.1	32.3	33.2	34.6	−1.2	−1.4
15	32.8	33.4	35.7	36.4	−0.6	−0.7

To further confirm compatibility with the assay, we evaluated the effect of using eNAT on assay analytical sensitivity. Contrived NP specimens prepared in eNAT or VTM from previously characterized negative samples were spiked with various concentrations of SARS-CoV-2 positive-control material (SeraCare) and run in triplicate (Table 3). We did not observe any difference in detection (6/6 targets detected) at 2× the LoD or the 250-copies/ml assay LoD. The same was true for specimens spiked with 125 copies/ml (6/6 targets detected); however, there was a loss in detection of SARS-CoV-2 in both eNAT (4/6 targets detected) and VTM (3/6 targets detected) at 25 copies/ml. To further evaluate the impact of eNAT on assay sensitivity, the mean *C_T_* values were compared for each concentration of the transport medium pairs. The *C_T_* values did not show statistically significant differences (*P > *0.05). Therefore, eNAT exhibits equivalent performance for the detection of SARS-CoV-2 relative to VTM and is compatible for use with the Cepheid Xpert Xpress SARS-CoV-2 assay.

**TABLE 3:** Effect of eNAT on analytical sensitivity of the Cepheid Xpert Xpress SARS-CoV-2 assay^*a*^

SARS-CoV-2 concn (cp/ml)	Medium	*C_T_* value						Mean *C_T_*		No. of positive targets
		E gene			N2 gene			E gene	N2 gene	
		1	2	3	1	2	3			
500	VTM	34.2	35.2	35.8	37.6	38.7	38.5	35.1	38.2	6/6
eNAT	34.2	34.3	35.0	38.0	37.2	37.8	34.5	37.6	6/6
250	VTM	36.7	37.1	35.4	38.0	38.1	39.9	36.4	38.7	6/6
eNAT	35.7	35.4	35.6	38.7	38.2	37.8	35.6	38.2	6/6
125	VTM	37.2	36.1	36.2	41.4	39.1	39.3	36.5	39.9	6/6
eNAT	36.8	35.3	36.5	40.8	40.2	40.8	36.2	40.6	6/6
25	VTM	ND	38.6	ND	ND	40.6	41.1	3/6
eNAT	38.6	ND	37.6	ND	39.9	42.0	4/6

a Abbreviations: SARS-CoV-2, severe acute respiratory syndrome coronavirus 2; cp, copies; *C_T_*, cycle threshold; VTM, viral transport medium; ND, not detected.

## DISCUSSION

Challenges surrounding biosafety and specimen stability have limited COVID-19 diagnostics in rural and remote regions. A pragmatic approach for communities with limited access to testing may be to implement transport media capable of inactivating SARS-CoV-2, enabling safe movement of specimens from the point of collection to processing in a centralized laboratory. In addition, a transport medium with nucleic acid-stabilizing properties over a wide range of temperatures (4 to 35°C) can extend testing access to regions that lack well-developed transportation networks. Even with the potential to expand testing to underserved populations, timely access to diagnosis in health systems with fragile specimen-transport logistics remains problematic. Therefore, a transport medium capable of viral inactivation and nucleic acid preservation has the potential to support molecular assays at POC and can, in turn, provide earlier detection of SARS-CoV-2, leading to improvements in case management and contact tracing.

Transport medium currently implemented (e.g., VTM, saline, and PBS) for the collection of swabs can maintain the viability of human coronaviruses for several days, including SARS-CoV-2 (5, 16–18). To reduce risk to personnel associated with the collection, transport, and processing of specimens, it is necessary to use a transport medium capable of viral inactivation. Our plaque reduction studies demonstrated high titers of SARS-CoV-2 to be inactivated within 10 min of incubation with eNAT. These findings are in support of those previously described that have shown inactivation of SARS-CoV-2 following 2 min (5) and 10 min (6) of incubation with eNAT. In these studies, the ratio of eNAT to virus varied from our conditions. However, under all test conditions with reagent-to-virus ratios of equal volume or favoring eNAT (10:1, 5:1, and 3:1), none had virus detectable in titration. In contrast, Welch and colleagues (6) demonstrated that for conditions where eNAT is diluted 1:3, eNAT concentrations are not sufficient to inactivate SARS-CoV-2. Although specimen dilution of eNAT is an important consideration, Copan collection devices are available in 1-ml and 2-ml eNAT volumes with flocked swab volume uptake of ∼142 μl (19). Therefore, when using the device as described by the manufacturer, the specimen would not dilute the reagent to concentrations suboptimal for SARS-CoV-2 inactivation.

We observed both eNAT and PBS to preserve the molecular detection of SARS-CoV-2 over a range of temperatures (4 to 35°C) throughout the 14-day evaluation period. To our knowledge, this is the first study evaluating the stability of SARS-CoV-2 RNA in eNAT; however, similar results at 4°C and room temperature (18 to 26°C) have been described for PBS previously (20–22). In contrast, storage time and temperature had a variable impact on SARS-CoV-2 detection for swabs stored in VTM, saline, or eSwab, especially when specimens were not refrigerated. These findings suggest the limited utility for VTM, saline, and eSwab for long-term transport of SARS-CoV-2 specimens in the absence of a cold chain.

Considerable variability exists in the literature related to SARS-CoV-2 RNA stability in alternative transport media. Factors associated with the preparation of contrived specimens for these stability studies, such as the volume of NP specimen spiked and host factors (nasal microbiota, immune status, etc.), are likely to contribute to this variability. Differences in the NP microbial communities have been reported among SARS-CoV-2-positve and -negative patients (23), and, unlike other studies (21, 22), we attempted to address this by using pooled SARS-CoV-2-positive patient material to represent a more clinically accurate specimen type. Furthermore, definitions as to what *C_T_* value increase is deemed significant for a loss in sensitivity or stability is not standardized across studies. We based our interpretation of a loss of sensitivity on the precision of the assay utilized for the stability studies. Our cutoff (>1 *C_T_* value increase) was more conservative than those of other studies (>2 or 3 *C_T_* value increase) (20, 21). Nonetheless, our findings suggest that eNAT offers greater stability for long-term storage/transport, even in the absence of a cold chain, than VTM, saline, and eSwab.

Current guidance by the WHO and U.S. Centers for Disease Control and Prevention requires testing of clinical specimens to be carried out in a BSL-2 setting. An exception to this is POC testing or near-POC, where biosafety guidelines allow testing to be performed outside a biological safety cabinet if appropriate precautionary measures are in place. Chemical inactivation at the time of specimen collection by the transport medium is the most practical for POC workflows. Immediate chemical inactivation eliminates infectious aerosols or droplet generation and, thus, reduces some of the operational requirements needed for safe handling of infectious respiratory samples at the POC. Of additional benefit, chemical inactivation does not require new equipment or cause delays in processing (e.g., heat inactivation). Although viral inactivation is not mandated for POC testing, eNAT can minimize operational requirements (e.g., additional personal protective equipment or splash shields) needed, ultimately improving workflow and safety for COVID-19 molecular diagnostics at POC.

In 2010, the WHO endorsed GeneXpert MTB/RIF for the rapid diagnosis of tuberculosis, leading to a massive scale-up of Xpert worldwide. Many high-burden countries have adopted a “hub-and-spoke” model for scale-up where Xpert instruments are placed in higher-level facilities with adequate infrastructure (e.g., security and stable power), known as the hub, which receive specimens from several lower-level health facilities (spokes). With this model, Xpert testing services have increased access to rapid and more sensitive diagnostic testing for patients who present to lower-level health facilities in underserved areas. As eNAT demonstrated compatibility with the Xpert Xpress SARS-CoV-2 assay, pairing this specimen transport device with existing Xpert systems and infrastructure can support the expansion of COVID-19 diagnostics in underserved communities.

Our study has limitations. Diluting out the cytotoxic effect of eNAT on Vero-E6 cells resulted in a higher LoD (500 PFU/ml rather than 50 PFU/ml) for the assay. In previous studies, the removal of eNAT from treated SARS-CoV-2 was performed (e.g., buffer exchange method or spin column filtration) before the addition of virus to plaque reduction assays (5, 6). These cytotoxic mitigating techniques significantly improved assay sensitivity, enabling the observation of complete inactivation of high titer virus (>10^7^ PFU/ml) at 5:1 reagent-to-virus dilution (5). Although these findings do not allow us to conclude complete inactivation of SARS-CoV-2 in our studies, it is likely that with improved assay sensitivity, further reduction in viral titers would have been observed.

Another limitation is that the use of contrived specimens throughout the studies may have underestimated the effect of possible RT-PCR inhibitors (i.e., bacterial and immune products), as these would have been diluted out during sample preparation. However, unlike the other transport medium evaluated, it is unlikely that the stability findings of eNAT would have been impacted by this dilution effect due to its protein denaturation and bacterial inactivation properties. As a result of limited reagent availability and institutional allocations, we were unable to include additional VTM-eNAT sample pairs in the Xpert Xpress SARS-CoV-2 accuracy studies. Therefore, conclusions from our studies are based on a small number of NP matrices and may not be generalizable to all NP specimens.

The current study solely focused on the performance of eNAT and its compatibility with the Cepheid Xpert Xpress SARS-CoV-2 assay. Although our findings suggest that eNAT can be used for COVID-19 diagnostics at the POC, additional evaluation is required for laboratories using other nucleic acid amplification assays to ensure compatibility. Lastly, in-field validation studies of the performance of eNAT with the Xpert Xpress SARS-CoV-2 assay are needed to confirm the findings of this study.

In conclusion, we investigated eNAT as an alternative transport medium for the collection of swabs for SARS-CoV-2 testing. Our findings suggest that eNAT can inactivate SARS-CoV-2 and can maintain specimen stability for an extended time, even in the absence of a cold chain (i.e., 14 days at 35°C). Improvements in biosafety and specimen stability can support the collection of specimens in the community and transport to BSL-2 laboratories, ultimately eliminating the challenge of the patient needing to travel to testing sites. However, for many communities worldwide, delays in specimen transport to centralized testing centers is common, translating to suboptimal turnaround times for test results. As eNAT can inactivate SARS-CoV-2 and is compatible with the Cepheid Xpert Xpress SARS-CoV-2 assay, COVID-19 diagnostics at the POC becomes possible. Therefore, findings from this study suggest that eNAT represents a promising mechanism to improve access to COVID-19 diagnostic testing for communities with limited health care access.
